# Solar powered oxygen systems in remote health centers in Papua New Guinea: a large scale implementation effectiveness trial

**DOI:** 10.7189/jogh.07.010411

**Published:** 2017-06

**Authors:** Trevor Duke, Ilomo Hwaihwanje, Magdalynn Kaupa, Jonah Karubi, Doreen Panauwe, Martin Sa’avu, Francis Pulsan, Peter Prasad, Freddy Maru, Henry Tenambo, Ambrose Kwaramb, Eleanor Neal, Hamish Graham, Rasa Izadnegahdar

**Affiliations:** 1 Center for International Child Health, University of Melbourne and MCRI, Melbourne, Australia; 2 School of Medicine and Health Sciences, University of PNG, Taurama Campus, NCD, Papua New Guinea; 3 Goroka General Hospital, Eastern Highlands Province, Goroka, Papua New Guinea; 4 Mt Hagen General Hospital, Western Highlands, Mount Hagen, Papua New Guinea; 5 Wabag Hospital, Enga Province, Wabag, Papua New Guinea; 6 Mendi Hospital, Southern Highlands Province, Mendi, Papua New Guinea; 7 School of Medicine and Health Sciences, University of PNG, Taurama Campus, NCD, Papua New Guinea; 8 AusTrade Pacific, Sydney, Australia; 9 AusTrade Pacific, Port Moresby, Papua New Guinea; 10 Health Facilities Branch, National Department of Health, Papua New Guinea; 11 Bill and Melinda Gates Foundation, Seattle, Washington, USA

## Abstract

**Background:**

Pneumonia is the largest cause of child deaths in Papua New Guinea (PNG), and hypoxaemia is the major complication causing death in childhood pneumonia, and hypoxaemia is a major factor in deaths from many other common conditions, including bronchiolitis, asthma, sepsis, malaria, trauma, perinatal problems, and obstetric emergencies. A reliable source of oxygen therapy can reduce mortality from pneumonia by up to 35%. However, in low and middle income countries throughout the world, improved oxygen systems have not been implemented at large scale in remote, difficult to access health care settings, and oxygen is often unavailable at smaller rural hospitals or district health centers which serve as the first point of referral for childhood illnesses. These hospitals are hampered by lack of reliable power, staff training and other basic services.

**Methods:**

We report the methodology of a large implementation effectiveness trial involving sustainable and renewable oxygen and power systems in 36 health facilities in remote rural areas of PNG. The methodology is a before–and after evaluation involving continuous quality improvement, and a health systems approach. We describe this model of implementation as the considerations and steps involved have wider implications in health systems in other countries.

**Results:**

The implementation steps include: defining the criteria for where such an intervention is appropriate, assessment of power supplies and power requirements, the optimal design of a solar power system, specifications for oxygen concentrators and other oxygen equipment that will function in remote environments, installation logistics in remote settings, the role of oxygen analyzers in monitoring oxygen concentrator performance, the engineering capacity required to sustain a program at scale, clinical guidelines and training on oxygen equipment and the treatment of children with severe respiratory infection and other critical illnesses, program costs, and measurement of processes and outcomes to support continuous quality improvement.

**Conclusions:**

This study will evaluate the feasibility and sustainability issues in improving oxygen systems and providing reliable power on a large scale in remote rural settings in PNG, and the impact of this on child mortality from pneumonia over 3 years post–intervention. Taking a continuous quality improvement approach can be transformational for remote health services.

Pneumonia is the largest cause of child deaths in Papua New Guinea (PNG), as it is globally [1,2]. Hypoxaemia is the major complication causing death in childhood pneumonia and is highly prevalent in highlands region of PNG. In provincial hospitals in PNG the mortality rate for pneumonia and severe pneumonia are 5% and 10%, respectively [2]. In previous studies in PNG we showed that improved oxygen systems, which include a reliable source of oxygen therapy using concentrators, and pulse oximetry for detection of hypoxaemia, can reduce mortality from pneumonia by up to 35% [3]. Hypoxaemia occurs in many other common conditions: bronchiolitis, sepsis, asthma, tuberculosis, HIV–related lung disease, malaria, perinatal problems, trauma, obstetric emergencies, and chronic respiratory and cardiac disease in adults and children [4–7]. A reliable oxygen system is therefore a critical minimum input for adequate health care in all settings where such emergencies present and such conditions are managed.

However, in PNG and in low and middle income countries throughout the world, improved oxygen systems have only been implemented where it is relatively easy to do so; in larger provincial or referral hospitals with adequate power supplies and staff capacity [3,8,9]. In these countries, including those with the largest shares of the global burden of childhood pneumonia deaths such as Ethiopia and PNG, a high proportion of the population live in rural areas, where access to urban centers and referral health care facilities is limited or non–existent. Improved oxygen systems have not been implemented at scale in such settings, and do not reach smaller rural hospitals or district health centers that serve as the first point of referral for acute illness. Many children with severe pneumonia present to these small rural hospitals and district health centers where hypoxaemia often goes undetected or untreated. Barriers to improving oxygen systems in these settings include the lack of adequate power, uncertain long–term reliability of equipment in remote settings, logistics of implementation, lack of preventive maintenance, lack of recognition of the importance of oxygen or identification of hypoxemia using pulse oximetry, limited training of health workers in the recognition of hypoxaemia, and lack of financial investment in quality health services in rural and remote areas. Oxygen concentrators have previously been run using solar power, but only on a small scale in a single health facility [10,11].

We report the steps needed to implement basic oxygen systems in 36 health facilities in remote rural areas of PNG. The decisions and steps involved included: defining the criteria for facilities where such an intervention is appropriate, assessment of power supplies and power requirements, the optimal design of a solar power system that can address both oxygen equipment load and for other essential health facility equipment, specifications for oxygen concentrators and other oxygen equipment that will function in remote and tropical environments, implementation logistics in remote settings, the role of oxygen analyzers in monitoring oxygen concentrator performance, the engineering capacity required to sustain a program at scale, clinical guidelines and training on oxygen equipment and the treatment of children with severe respiratory infection and other critical illnesses, program costs, and measurement of processes and outcomes to inform continuous quality improvement.

These steps to scaling up oxygen systems in the most challenging environments have not previously been actioned in a large field trial. We describe this model of implementation in the hope that other countries will build on this.

## The project

Previous research has been conducted to understand the epidemiology of hypoxaemia in PNG [4,12,13], the quality of care provided in rural and district hospitals [14], and the effect of oxygen on case fatality rates from pneumonia [3]. What has not been demonstrated until now is the sustainable implementation at large scale in the most difficult environments, and the steps required to achieve this. This project is funded by the Bill and Melinda Gates Foundation and other partners. Project funding, which also included support for trialing the implementation of oxygen concentrators in 12 urban hospitals in Nigeria [15], was provided in 2014. Preparatory work for this project occurred in 2014 and 2015.

## METHODS

### The process for identifying suitable facilities

Criteria for selecting suitable health facilities was determined by wide stakeholder consultation between representatives of the National Department of Health, provincial health, nurses and doctors, administrators and technicians. Broad principles for selection were set out and suitability was ascertained by on–site assessments and review of the available routine data. Selection of participating health facilities was on the basis of clinical need and the capacity for benefiting that community. The need for sources of oxygen and power are high for health facilities that are located within large population areas (even if that population is dispersed over a wide area) where the prevalence of pneumonia and other acute respiratory infections is high, and where access to a provincial referral hospital is limited or transportation is likely to be hazardous for seriously ill patients. Other principles for health facility selection were that the health facility had difficulty gaining access to a reliable source of oxygen; that the facility was open and functioning; and staff were motivated to participate. In PNG while some primary health centers are closed or not functioning, many, particularly district health centers fulfilled these criteria, are functioning to the best of their capacity and have staff who are enthusiastic and committed.

Baseline data were collected on all proposed health facilities. This involved routine recording of admissions, deaths and referrals for the previous 3 years (**Table 1**).

**Table 1:** Baseline data in 36 health centers and district hospitals

Year	Paediatric admissions	Paediatric deaths	CFR (%)	Pneumonia	Pneumonia deaths	Pneumonia CFR (%)	Births	NND	NMR (per 1000)	Children referred out
2014	8511	309	3.63	3510	146	4.16	10933	98	9.0	687
2013	7193	236	3.28	2849	125	4.39	10329	88	8.5	695
2012	5104	216	4.23	2447	86	3.51	10284	84	8.2	791
**Total**	**20808**	**761**	**3.66**	**8806**	**357**	**4.05**	**31546**	**270**	**8.6**	**2173**

NND – neonatal deaths, CFR – case fatality rate

Data were also collected on characteristics of the health services: the number of health personnel, levels of training and recent continuing education, health facility bed capacity for children, availability of oxygen equipment, existing power sources, security issues at the health facility, and essential medicines, equipment, commodities and procedures for infection control, and laboratory tests.

### Assessment of power supplies and power requirements

We have previously assessed the power supplies in five district hospitals using an Electrocorder [14]. This demonstrated that even in facilities that are connected to mains power, power supplies are erratic, with many outages, fluctuations and surges. These abrupt changes in power have the high likelihood of damaging oxygen concentrators and other equipment, and may be one of the key reasons for concentrator failure in previous studies. In this current field trial we did not reproduce Electrocorder readings as the health facilities included were at an even lower level than the previously assessed 5 district hospitals, and were either without mains power, with known unreliable power, or with an alternative source of renewable power, such as hydropower. If a health facility only had a diesel backup generator this was not considered sufficient, as the costs, availability and logistics of diesel fuel are an obstacle to using electrically powered equipment.

### The optimal design of a solar power system

Considerations in the design of the solar panel included the efficiency of the system, the peak sun hours for the specified tilt angle (4.53 hours), a 25% oversupply of power, and the total daily energy demand. The system was designed to supply 11.44kWh/d daily average load, using eighteen 240W panels. This was based on the power requirements for a rural health facility for treating common childhood illnesses [16]. A battery backup system was designed to provide 3 days autonomy at 80% depth of discharge. Other solar equipment calculations are included in **Online Supplementary Document(Online Supplementary Document).**

### Specifications for oxygen concentrators and other oxygen equipment that will function well in remote environments

The choice of oxygen equipment was based on over a decade of trial and error, in PNG and in other countries [8,17–20]. The principles are that concentrators needed to be robust, standardized, and able to function well at high temperature and high humidity, and have a relatively low power requirement. Concentrator specifications follow the now published WHO guidelines for oxygen concentrators [21]. We use Airsep Elite 5 l/min concentrators (Chart Industries, New York NY, USA) and Lifebox pulse oximeters (Lifebox, London, UK), both of which have been successfully used in larger hospitals in previous field trials in PNG and other developing countries. Busy district hospitals are receiving three concentrators, and smaller health centers are receiving two. Theoretically, several patients can be treated at any one time using a concentrator using a flow meter assembly which splits flow to several patients (Sureflow meter, Airsep FM069–1, Chart Industries, Galveston USA). Typically two patients at any one time can be safely managed using one concentrator, even if they are each severely hypoxic and requiring standard flow rates of 2 L/min. Sometimes very unwell children will require higher flows in the initial treatment period and may need a single concentrator [19,22].

### Installation logistics

This project involves implementation teams and provincial monitoring teams. Implementation consisted of several skilled teams of 4–5 workers. The team leaders were trained in installing the solar power system, and in commissioning and use of oxygen concentrators. Large transport logistics were required to haul equipment from wharf up the PNG highlands highway, and smaller vehicles were required on unmade feeder roads to remote health centers. The installations were preceded by site visits, to discuss with the community and health workers, to ensure facilities fulfilled the criteria for inclusion, and to plan the siting of solar panels for maximal effectiveness. The installations typically took 2–3 days in each center, occurring between March and October 2016.

The implementation teams followed up to assess whether the equipment was being properly used and cared for.

### Monitoring oxygen concentrator performance using oxygen analysers

Health care workers, particularly in remote settings, have an important role in maintaining equipment. For decades many oxygen concentrator projects have failed the test of even medium term sustainability because of technical faults that were detected late or not at all, leading to entire oxygen system failure. In most health facilities throughout the world qualified engineers do not monitor equipment regularly (as engineers may be stationed at a major hospital rather than a district hospital or health center). Therefore preventative maintenance of oxygen concentrators (apart from occasional external filter cleaning) is limited or non–existent. Use of simple oxygen analyzers (Maxtec O_2_ analyzer, Maxtec, Utah USA) by health care workers to monitor the performance of concentrators (fraction of oxygen produced, to ensure it is >85%), and the flow rate, and training health workers to understand the meaning of alarms on the concentrator will enable early detection of developing faults or parts wearing, and early communication with provincial engineers. Given the limits of current concentrator technology this is a key to addressing the problems of all previous research in concentrators in low resource settings. We taught all health staff how to use oxygen analyzers and provided one for each health facility.

### The engineering capacity required to sustain a program at scale

Previous oxygen projects have had centralized engineering capacity, but these have floundered because technicians change roles, move into the private sector, or because of over–reliance on one or two busy persons. In this project we have decentralized the technical capacity as much as possible, by optimizing what nurses in health centers can do in preventative maintenance and monitoring of equipment performance, and training provincial and district level technicians, and having spare parts available in provincial locations which are at most 2–5 hours’ drive away.

### Clinical guidelines and training on oxygen equipment and the treatment of children with severe respiratory infection and other critical illnesses

We use the WHO guidelines for the Clinical Use of Oxygen in Children [22], and the WHO Hospital Care for Children for training [23]. The training is practical, initially workshop based, and repeated on several occasions and reinforced on health center visits by the provincial pediatricians. The training combines clinical and technical teaching and experience, and the messages are heard in more than one forum and more than one way – direct facilitator led teaching, peer and facilitator supervised practical examples, breaking complex tasks and skills into component parts and modeling each component, and presenting a holistic approach to the management of sick children. Training covers an understanding of hypoxaemia in pneumonia, other acute respiratory infections and non–respiratory diseases, respiratory clinical signs, principles and practical use of pulse oximetry, oxygen concentrators and how they work, preventative maintenance and trouble–shooting, use of oxygen analysers for assessing performance of concentrators, safe use of oxygen therapy in newborns, resuscitation, and the concept and practical aspects of continuous quality improvement. The curriculum is based on the WHO guidelines [22].

For many health care workers, the health technologies we introduce are new and sometimes daunting. Oxygen concentrators, like all technologies, may fail because people cannot understand them, or are wary of them, or cannot competently perform the practical tasks of checking and maintenance. A considerable part of this project on the sustainability of better oxygen systems in remote low resource settings involves a novel technology–clinical interface. Until now, these health care settings have been a technology–free zone and the integration of technology into clinical medicine in these settings is unproven and experimental. The elements of a successful technology–clinical interface, if it can be achieved, need to be better understood. If health care workers and technicians have not physically performed a given procedure during training, it is not likely they can do it in their own health care setting (eg, changing oximeter probes, replacing oximeter batteries, replacing concentrator filters, testing concentrator alarms and using an oxygen analyzer). Clinical and technical training tools are available at http://www.hospitalcareforchildren.org/.

### Program costs

It is relatively inexpensive to provide oxygen concentrators, however solar power is expensive. The cost per health facility is approximately US$ 50 000, including oxygen and solar equipment, installation and logistics, and training. Over the course of the formal training, on–site support and continuous quality improvement, all health workers who manage children in these 36 facilities will have training and hands on assessment of skills.

## RESULTS

### Baseline data on logistics and health services

Of 36 health facilities originally proposed, 6 were unsuitable because of poor road conditions that would not allow a vehicle carrying equipment to access the health center (n = 3) or the health center was in a state of disrepair (n = 3). Six more health facilities which fulfilled the criteria were selected to replace these. The catchment population of these 36 health facilities was 1 223 755 in the last (2011) National census, out of a total PNG population of 7.013 million. The median number of beds for children was 10 (interquartile range IQR 5–13), and newborns was 4 (0–8). Only 10 of the 36 health facilities have doctors. The median number of nurses on staff was 4 (IQR 2–10), and 29 had one or more midwives (12 health facilities had only one midwife). Only 16 of the health facilities had a trained pediatric nurse, with the other 20 having general trained nurses. Community health workers were present in all health facilities, with the median number of 7 (IQR 3–17). Only 16 health facilities were connected to mains power, 21 had a petrol generator. Running water was available in 30 health facilities (83%). Most health facilities had simple antibiotics for the treatment of pneumonia, either amoxycillin (n = 33) or penicillin (all health facilities). 34 had vaccine refrigerator, and most were stocked with vaccines: measles vaccine (n = 34), Pentavalent (32), but BCG was less available (25 health facilities). All but one had infant weighing scales and safe sharps disposal, but a minority (10) could check blood glucose, hemoglobin (10), or had x–ray facilities (7). Malaria tests were available in 28 and sputum smear for tuberculosis in 20.

### Baseline data on outcomes

In the 36 participating health centers we gathered data on over 20 000 admissions in the 3 years prior to the installations (**Table 1**). Over 8000 admissions were for pneumonia. The overall case fatality rate was 3.7% and the case fatality rate for pneumonia was 4.1%. Just over 10% of admissions required referral to provincial hospitals. These baseline data were collected from health facility admission record books, which are historically meticulously kept by nursing staff in PNG.

A sample size calculation was based on the primary outcome of reducing pneumonia mortality – from 4% to 3%, with 90% power = 7295 in each arm. The study was also adequately powered to detect a different of 20% in overall mortality rate, and a 20% reduction in referrals to tertiary centers. Other outcomes are listed in **Table 2**.

**Table 2:** Outcomes, research questions, sources of data and specific metrics

	Outcome category	Research questions	Sources of data	Metrics
1	Deaths from pneumonia; overall pediatric deaths in health facility	Is there a difference in pneumonia case fatality rates and overall pediatric CFR from pre to post implementation of improved oxygen therapy and solar power?	Admission record books	**Pneumonia case fatality rate**: Pneumonia deaths / pneumonia admissions (%). **Paediatric case fatality rate**: Paediatric deaths / all pediatric admissions (%).
2	Referral / transfer	Is there any difference in referral rates from pre to post intervention?	Admission record books	Paediatric transfers / all pediatric admissions (%).
3	Patient characteristics and response to oxygen therapy – effectiveness of oxygen therapy using the method we have designed to treat hypoxaemia	What are the conditions associated with hypoxaemia in remote rural health facilities? What is the response to oxygen therapy when oxygen is given using solar–powered oxygen concentrators? What is the duration of hypoxaemia in children managed in remote rural health facilities?	Standardised admission record data	Diagnoses associated with hypoxaemia (proportions) disaggregated for neonates and children >1 mo. Response to oxygen therapy (median change in SpO_2_ in the first 30 min; and proportion responded / not responded, ie, proportion with persisting SpO_2_<90%, or severe signs of respiratory distress 30 min after commencing oxygen). Days of oxygen therapy. Duration of hypoxaemia for neonates and children >1 month.
4	Maintenance of oxygen equipment	Are concentrators maintained well, are problems identified and is appropriate action taken? What proportion of concentrators undergo weekly maintenance and performance checking? What problems are identified? What proportion of concentrators are functioning well after 1, 2 and 3 years since installation?	Oxygen concentrator performance log–books. On–site checks on support and monitoring visits	What proportion of oxygen concentrators undergo weekly maintenance and performance checking? List of problems identified and action taken. Number / proportion of concentrators providing >85% oxygen and reliable flow rates as checked by oxygen analyzer at 1, 2 and 3 years.
5	Health workers knowledge and skill of oxygen therapy	What is the oxygen knowledge and skill of the health workers in remote health facilities? Does this improve with training and CQI	Oxygen competency tests – repeated measures	Repeating oxygen knowledge and skill tests at 12 monthly intervals.
6	Reliability, efficiency and adequacy of solar power	Solar power output (quantitative kW hours per day), adequacy of this power for running concentrators and other equipment needed by the health facility, and any problems identified.	Tristar 60 Amp controller	**Solar power output**: average kW hours per day produced by each solar system in the previous 6 mo. Occurrence of breakdown of solar output, such that concentrators are unable to function.
7	Training outcomes	What training was done and what are the perceived training needs?	Project records	Record of training courses conducted: formal; in–house as part of CQI
8	Sustainable processes	Is it sustainable in PNG to have concentrators run off solar power as the source of oxygen in remote areas?	Mixture of the above data sources, and documentation of relevant events, meetings and occurrences	How would we measure sustainability? **Equipment sustainability** (ie, proportion of concentrators and oximeters still functioning well at 1, 2, 3 years). **Energy sustainable** (proportion of solar power systems still functioning well at 1, 2, 3 years). **Health worker sustainability** (proportion with good oxygen knowledge and skills at 1, 2, 3 years). **Financial sustainability** (examples where national, provincial or local governments have invested in other facilities in a similar way). **Policy sustainability** (occurrence of a national oxygen policy that incorporates these principles). Documentation of other examples of engagement by policy makers.
9	Wider benefits	Does CQI in rural health facilities improve wider outcomes? Care seeking by parents? Health worker morale?	Above data and qualitative assessments	**Care seeking**: Number of children brought to health facilities (admission activity number per year for 3 years, compared with pre–intervention baseline data). **Women having confidence in the health services:** Number of babies born at health facilities (births per year for 3 years, compared with pre–intervention baseline data). **Qualitative assessment** of what health workers think of the oxygen project, whether and how it helps them in their work, what problems it may have resulted in, what could be done to help them better serve their communities, how they feel about their work, what their hopes are for their careers.

CQI – continuous quality improvement, CFR – case fatality rate, SpO_2_ – arterial oxygen saturation

### Assessing continuous quality improvement outcomes

Continuous quality improvement (CQI) involves regular monitoring and supervision by provincial supervisory teams; each team consists of the provincial pediatrician and a technician trained in oxygen and solar equipment. These reviews will occur every 4 months after implementation, will be carried out by the provincial teams and will include on–site training, collection of data on functioning of equipment, utilization, maintenance needs, and trouble–shooting of problems identified. There will be multiple assessments during these visits over the 3 years, including for example, whether nurses can accurately use oxygen analysers to effectively monitor the performance of concentrators, and whether the solar power capacity is sufficient for energy demand. The CQI approach involves feedback by the provincial monitoring teams – feedback is to the health center staff, the district and provincial authorities, and the National Department of Health. CQI outcomes also include whether interventions occurs in response to feedback, and the effect of these responsive interventions. An assessment of sustainability processes, including policy maker engagement, and the wider effects of this project on care seeking, health facility functioning and health worker morale is under way (**Table 2**).

## DISCUSSION

Over the past two decades mortality from childhood pneumonia has decreased significantly. While much effort has been put into vaccines and first line antibiotics for pneumonia, there is still a need for scaling up broader prevention and treatment initiatives. Even if severe pneumococcal infection could be markedly reduced by vaccination, realistically there will still be a myriad of respiratory viruses, particularly respiratory syncytial virus, and other viruses such as influenza, human metapneumo virus, parainfluenza; non–vaccine type pneumococci and *Haemophilus*; tuberculosis and other bacteria, which cause lower respiratory tract infections and fill up hospitals in low– and middle–income countries, as is the case in developed countries. The residual mortality, regardless of etiology will likely rest in those with more than a need for antibiotics alone. The population will likely be a more complex, comorbid, undernourished population where supportive care is increasingly important, including a role for oxygen therapy. The challenge of how to provide services in primary care and regional centers is not unique to PNG or to even to developing countries, the same drive to improve quality and reduce the need for referral is a focus in wealthy countries also. But the challenges are greater in rural, low income settings.

This implementation paper documents the steps to scaling up oxygen therapy in remote rural settings. Many steps are involved and this is a complex health care intervention. Previously oxygen has been thought of as an essential drug [24], but it is also an essential *service*. With the drive to increase facility births in low income, high mortality countries, health facilities have to reach a certain standard to signal to the community that they can provide a greater level of service than delivering at home – these essential services include power, clean water, oxygen, and infection control and prevention. As more and more births in countries like India, Ethiopia and Nigeria are happening in health facilities, there has been an ecological and foundational shift in care at these facilities. It is important to understand the importance of oxygen as a relatively simple yet essential service to maintain a minimum standard of quality in the redefined roles of primary health care and first referral facilities. Other investments, such as building sleeping quarters for staff to allow them to stay overnight, have to be prioritized also, and where oxygen, power, and running water fit into these priorities is a matter of judgement, but they all can be regarded as essential. PNG still has low rates of facility delivery (40%), but further increases will require explicit attention on ensuring that minimum services are in place close to where communities live.

We emphasize the importance of a holistic health systems approach, community engagement, and continuous quality improvement. To be sustainable and maximally effective such interventions should integrate into routine health system functioning, and the benefits must be cross–cutting for maximum adoption and effect. For example, there is limited value in installing solar power to run an oxygen concentrator if there is no light in the delivery room, as women will not want to deliver babies overnight. Lack of basic services effect the health center’s reputation and have adverse effects on community demand and care seeking (such as when an infant is unwell with pneumonia). However addressing such conditions, for example by providing power to run a light, and the vaccine refrigerator, and oxygen, can be transformational [16,25]. To be maximally efficient, such interventions should be based on a holistic and systematic approach to improving health service quality, with the provision of basic oxygen technology and adequate power as an entry point. Along with transforming a community’s perception of a health center, essential services like oxygen and a reliable power system, and training to match, can boost health workers confidence and morale.

PNG is an expensive place to do such work, as the terrain is difficult, and distances far. Many concentrator programs only report the oxygen concentrator costs, but we have previously shown that the implementation and training costs are at least as much again as the costs of concentrator and other oxygen equipment [1]. Sustainability and expansion of such programs require an accurate estimation of true costs and benefits, so that health administrators and governments can order priorities according to their needs and program according to their available resources. In a decentralized system where provinces and districts have to decide on their health spending priorities, this project leaves no doubt as to the full costs of such a health facility service upgrade. There are many health priorities for child health in the sustainable development era, including health facility quality improvement, and each have real costs that have to be weighed [26].

Over the next 3 years we will measure clinical effectiveness and what is required to sustain this program, from simple things like whether nurses can accurately use oxygen analysers to effectively monitor the performance of concentrators, to whether the solar power capacity is sufficient for energy demand. And we will monitor, beyond effectiveness in the field, the administrative and political steps required for decisions about involvement of other provinces in the program, and how such interventions can be seen as cross–cutting, and part of a greater whole – quality services at district hospitals and rural health centers. A serious problem for many such rural health services and primary health care workers in the past has been low expectations, and showing that appropriate and essential technology such as this can be effective and sustained may just raise expectations to the level needed in the era of sustainable development.

## CONCLUSIONS

This study will evaluate the feasibility and sustainability issues in improving oxygen systems and providing reliable power on a large scale in remote rural settings in PNG, and the impact of this on child mortality from pneumonia over 3 years post–intervention. Taking a continuous quality improvement approach may be transformational for remote health services.
