# Supplementary material for: Solar powered oxygen systems in remote health centers in Papua New Guinea: a large scale implementation effectiveness trial
Source: J Glob Health. 2017 Apr 23;7(1):010411. doi: 10.7189/jogh.07.010411 (PMC5441450; doi:10.7189/jogh.07.010411)
Supplement: Online Supplementary Document [file jogh-07-010411-s001.pdf]

Online Supplementary Document

Duke et al. Solar powered oxygen systems in remote health centers in Papua New Guinea: a large scale implementation effectivenss trial.
J Glob Health 2017;7:010411

Solar power and engineering calculations

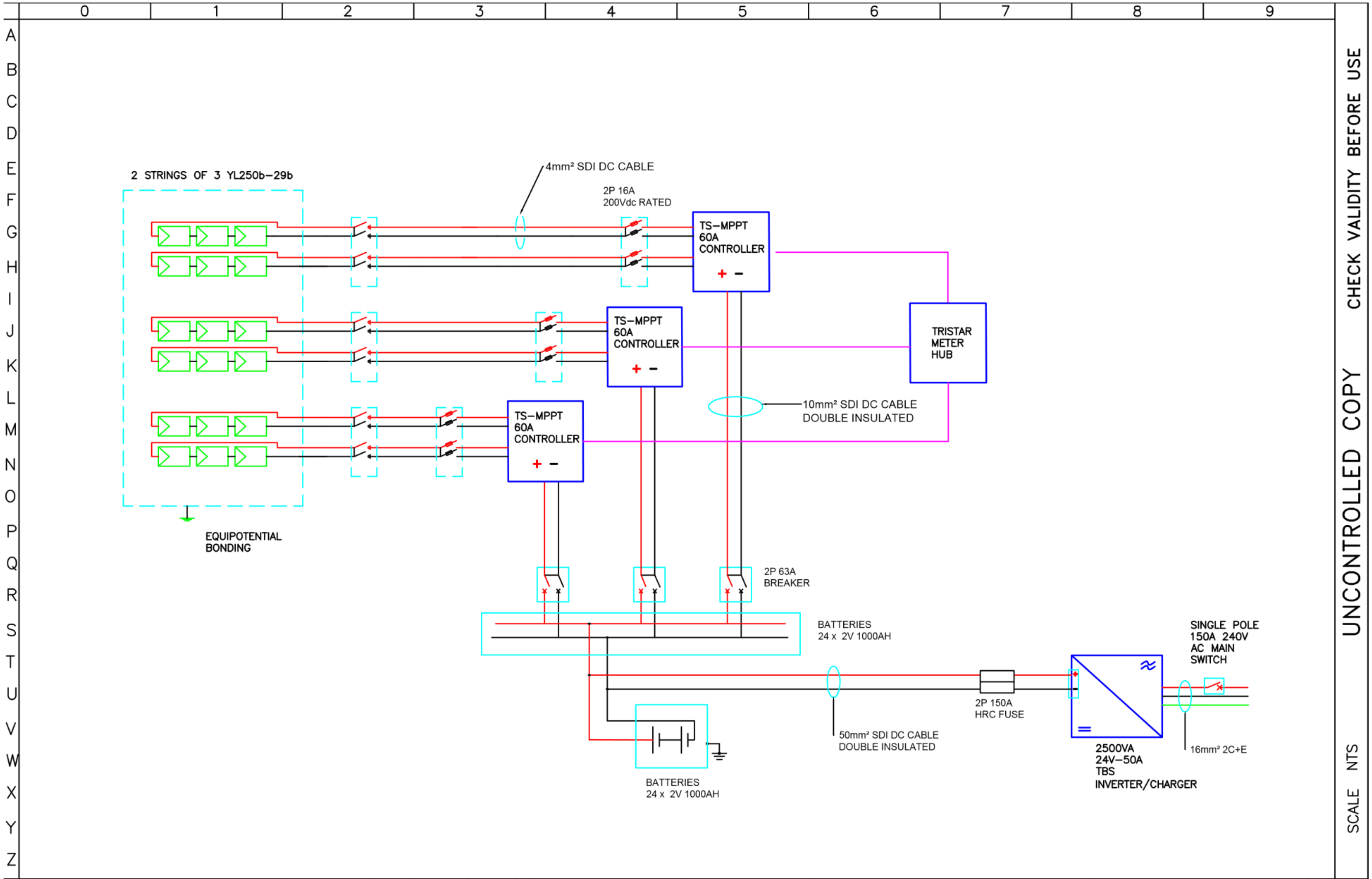

ISSUE	DETAILS OF AMENDMENTS	DATE	DRAWN	DESIGNED	APPROVED
04	REV 0				
03	NTS				
02					
	ORIGINAL	10–MAR–15	SC	KP	AP

CBS Power Solutions

CBS POWER SOLUTIONS FIJI
SHOP 1A DANIVA RD
VALELEVU, NASINU FIJI ISLANDS
p: +679 334 2428
f: +679 334 1425
e: amit@cbspowersolutions.com
w: www.cbspowersolutions.com

TITLE: PNG SCHEMATIC
4.5 kW OFFGRID SYSTEM

DOCUMENT NUMBER: ELEC_ OF

ISSUE: AA

A3

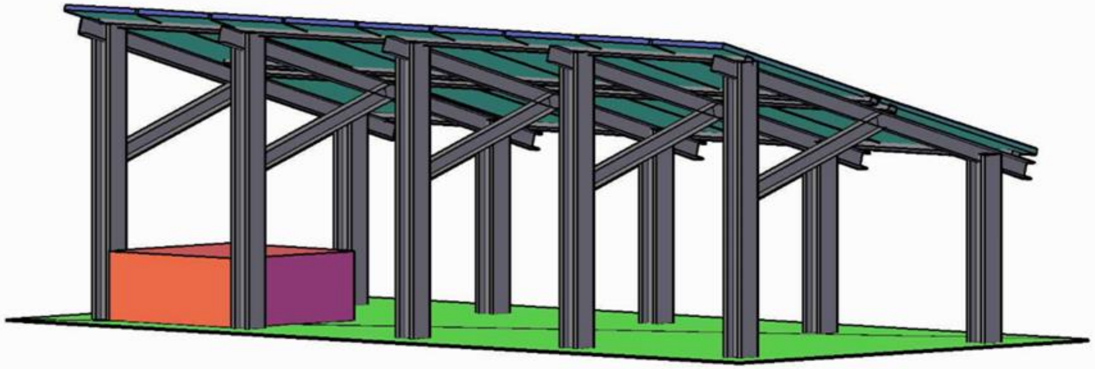

2 STRINGS OF 13 YL250P–29b PANELS

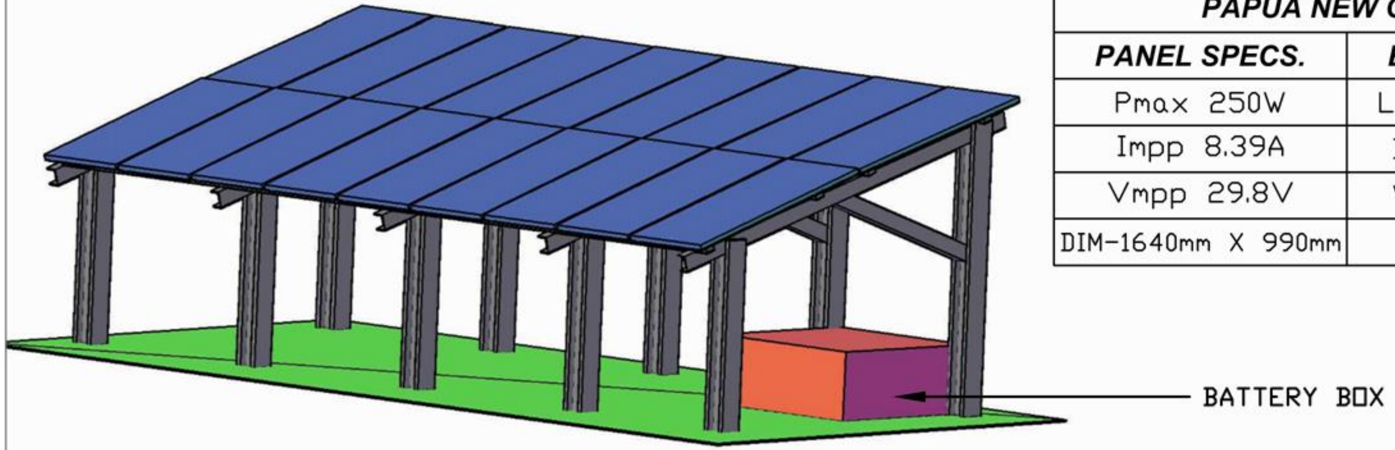

PAPUA NEW GUINEA - OFFGRID SYSTEM		
**PANEL SPECS.**	**BATTERY BOX**	**BATTERY**
Pmax 250W	LENGTH 1400mm	1000AH
Impp 8.39A	DEPTH 850mm	48V
Vmpp 29.8V	WIDTH 950mm	
DIM–1640mm X 990mm		

01	ALL MEASUREMENTS IN MM			
02	NTS			
03				
04				
05	ORIGINAL	02-03-14	SC	KP
ISSUE	DETAILS OF AMENDMENTS	DATE	DRAWN	DESIGNED

CBS Power Solutions

CBS POWER SOLUTIONS FIJI
SHOP 1A, DANIVA RD
VALELEVU, NASINU, FIJI ISLANDS
p: +679 334 2428
f: +679 334 1425
e: amit@cbspowersolutions.com
w: www.cbspowersolutions.com

TITLE
PNG PROJECT
PANEL STRUCTURE

4.5 kW OFFGRID SYSTEM

DOCUMENT NUMBER
PVSTRCTRE_01 OF 01

SYSTEM DESIGN & ENGINEERING CALCULATIONS

CIRCUIT 1

Description	Value	Unit
Load in Watts	1339	Watts
Days of Autonomy	3	Days
System Voltage	24	Vdc
**SOLAR MODULE SPECS**		
Maximum Power	250	Watts
Maximum Voltage	29.8	Volts
Maximum Current	8.39	Amps
Open Circuit Voltage	37.6	Volts
Short Circuit Current	8.92	Amps
Temperature Coefficient of P_{max}	-0.42	%/°C
Temperature Coefficient of V_{oc}	-0.32	%/°C
Temperature Coefficient of I_{sc}	0.05	%/°C
Power Tolerance f_{man}	-0/+5	W
**ENVIRONMENT CONDITIONS & LOSSES**		
De-rating factor for direct f_{dirt}	2	%
Peak Sun Hours	4.53	Hrs/day
Design Maximum Ambient Temperature	30	°C
Design Minimum Ambient temperature	17	°C
Site Maximum Ambient Temperature	30	°C

The Temperature derating factor is determined as follows:

$$f_{temp} = 1 - [\gamma \text{ x } (T_{cell.eff} - T_{stc})]$$

- *Cell temperature at standard test conditions 25°C*
- *Average daily cell temperature in °C*
- *Power temperature coefficient per degree Celsius*
- *Temperature de-rating factor (dimensionless)*

$T_{cell.eff}$ = Ta + 25°C = 28°C + 25°C =53°C f_{temp} = 1 –

[0.42/100 x (53– 25)] = **0.882**

The Temperature derated Output of module is determined as follows:

$$P_{mod} = P_{stc} \text{ x } f_{man} \text{ x } f_{temp} \text{ x } f_{dirt}$$

- *De-rating factor for dirt (dimensionless)*
- *De-rating factor for temperature (dimensionless)*
- *Manufacturing Tolerance (as per manufacturers datasheet)*
- *Rated Module output under Standard Test Conditions (STC)*
- *De-rated Power output of the module in Watts*

P_{mod} = 250 x 1.02 x 0.882 x 0.98 = **220.41Watts**

To determine the number of modules in the array to meet the daily design energy demand is calculated as follows:

$$N = (E_{tot} \times f_o) / (P_{mod} \times H_{tilt} \times \cap_{pvss})$$

Efficiency of the PV Sub-system (dimensionless)

Peak Sun Hours for the specified tilt angle

De-rated power output of the module in Watts

Oversupply co-efficient (dimensionless) _ we have designed the system with 25% oversupply

Total Daily design Energy Demand

Number of Modules required in the array

Etot = (290W x2x24Hrs x3dys/7dys)+(200Wx8Hrs)+(9Wx16x5Hrs)+(15Wx24Hrsx3dys/7dys)+(3000Wh) Etot = 11440 Wh/day

N = (11440Wh x 1.25) / (220.41 x 4.50 x 0.80) = **18.02**....round off to **18 panels**

Since our design is based on MPPT analysis, we will look at an MPPT controller which can handle 18x250Wp=4500Wp

Controller chosen: controller 1 - 1 x 60A Tri-Star MPPT

Controller 2 - 1 x 60A Tri-Star MPPT

Controller 3 - 1 x 60A Tri-Star MPPT

Array Configuration:

6 panels per controller

2 strings of 3 panels in series per controller (See schematic attached)

The controllers will have synchronized charging to the battery with a meter Hub.

Battery Calculations

To determine the size of Battery Bank to meet the daily design energy demand is calculated as follows:

$$C_x = (E_{tot} / AV_{dc}) \text{ x } (T_{aut} / D.O.D)$$

Total number of days of autonomy required in days

System Average Voltage in 24 Volts

Total energy requirement in Watt hours

Battery Bank Capacity at a certain discharge rate, x

Battery Bank at 80% depth of discharge and 3 days back up:

C_{10} = (11440 / 24) x (3 /0.8) = **1787.5 AH Bank**

System Configuration:

18 x 250W Panels

1800Ah@24V Battery bank

Cable sizing and Voltage drop calculations

-Length of DC cable $L_{DC\ cable} = 10m$
-Maximum power point current of panel $I_{MP} = 8.39A$
-Maximum power point voltage of array $V_{Array\ max}$.....Refer to calculation below

$$V_{Array\ max} = No. of\ panels\ \{V_{mp} - [\gamma_{Vmp}\ (T_{min} - T_{STC})]\}$$

$V_{Array\ max} = 3\ \{29.8 - [0.125(50°C - 25°C)]\}$ (At lower temperature since voltage would be higher)

$$V_{Array\ max} = 80.025\ V$$

Determining cross sectional area of the array cable

$$A_{DC\ cable} = \frac{2\ x\ L_{DC\ cable}\ x\ I_{MP}\ x\ \rho}{\%V_{drop}\ x\ V_{Arraymax}}$$

$A_{DC\ cable} = 2\ x\ 10\text{m}\ x\ 8.39\ x\ 0.0183 / 0.01\ x\ 80.025$ ....(1% voltage drop assumed)

$A_{DC\ cable} = 3.83mm^2$ ……….. $\boldsymbol{4mm^2}$ ***SDI cable would be suitable***

Determining cross sectional area of Battery charging cable

$$A_{DC\ cable} = \frac{2\ x\ L_{DC\ cable}\ x\ I_{dcbat}\ x\ \rho}{\%V_{drop}\ x\ V_{Batsys}}$$

$A_{DC\ cable} = 2\ x\ 5\text{m}\ x\ 60\ x\ 0.0183 / 0.01\ x\ 24$ ....(1% voltage drop assumed)

$A_{DC\ cable} = 45.75$ ……….. $\boldsymbol{50mm^2}$ ***PVC cable would be suitable***

Determining cross sectional area Inverter DC cable

$$A_{DC\ cable} = \frac{2\ x\ L_{DC\ cable}\ x\ I_{dcinv}\ x\ \rho}{\%V_{drop}\ x\ V_{inverterdc}}$$

$A_{DC\ cable} = 2\ x\ 5\text{m}\ x\ 105\ x\ 0.0183 / 0.01\ x\ 24$ ....(1% voltage drop assumed)

$A_{DC\ cable} = 80.08mm^2$ ……….. $\boldsymbol{80mm^2}$***PVC Battery cable chosen***

Inverter Selection calculations

AC Load

(16 x 9W LED lights)+(2x290W Oxy Concentrator)+(200W desktop)+(1x15W Oximeter)+(650kWh/yr) =**1339 W**

The oxygen concentrator will have a surge for very short period of time, so we need to make sure that the inverter chosen can supply that surge for a short period, usually 1 second.

In this case the inverter size chosen is a **2500VA 24V TBS Inverter**. Refer to attached datasheet for specs.

Protection Equipment Sizing

DC breakers:

PV breaker sizing

Rated PV breaker Voltage

$$V_{OC\ arraymax} = No.\ of\ panels\{V_{OC} - [\gamma_{Voc}(T_{min} - T_{STC})]\}$$

$$V_{OC\ arraymax} = 3\left\{37.6 - \left[\frac{0.32}{100} x\ 37.6(50 - 25)\right]\right\} = 104.96V$$

$$V_{OC\ arraymax} = V_{PV\ Breaker}$$

$V_{PV\ Breaker} = \mathbf{103.78}V$ …**Approx 125Vdc rated PV Breaker**

Rated PV Breaker Current

$$I_{Pv\ Breaker} = 1.25\ x\ I_{sc\ array}$$

$$I_{Isolator} = 1.25\ x\ 8.92A$$

$I_{Isolator} = 11.15$ … …**Approx 16A rated PV breaker required**

Battery Charging Breaker

Rated voltage to be 24Vdc as per battery bank voltage

Rated current= 1.25% x Rated controller current= 1.25% x 60A= **75A** Breaker

Inverter DC Side Breaker

For a 2500VA 24V TBS Inverter:

Inverter surge power/system voltage=5500W/24V=229.17A....**250A HRC Fuse**

Inverter AC Side Breaker

Inverter AC surge power/AC system voltage=5500W/240=22.9.....**32A Main Switch**

Yearly Average Energy Yeild

Overall system efficiency

$$f_{system} = f_{inv}\ x\ f_{voltdropACDC}\ x\ f_{dirt}\ x\ f_{temp}\ x\ f_{man\ tol}$$

$$= 0.93\ x\ 0.98\ x\ 0.98\ x\ 0.91\ x\ 1$$

$$= \mathbf{0.812......81.2\%}$$

Expected yearly output of the system

$$E_{avg} = No.\ of\ panels\ x\ P_{mod\ derated}\ x\ peak\ sun\ hours\ x\ 366\ days\ x\ f_{system}$$

$$= 18\ x\ 220.41W\ x\ 4.53\ x\ 365\ x\ 0.812$$

$$= \mathbf{5.33}\ \boldsymbol{MWh/year}$$

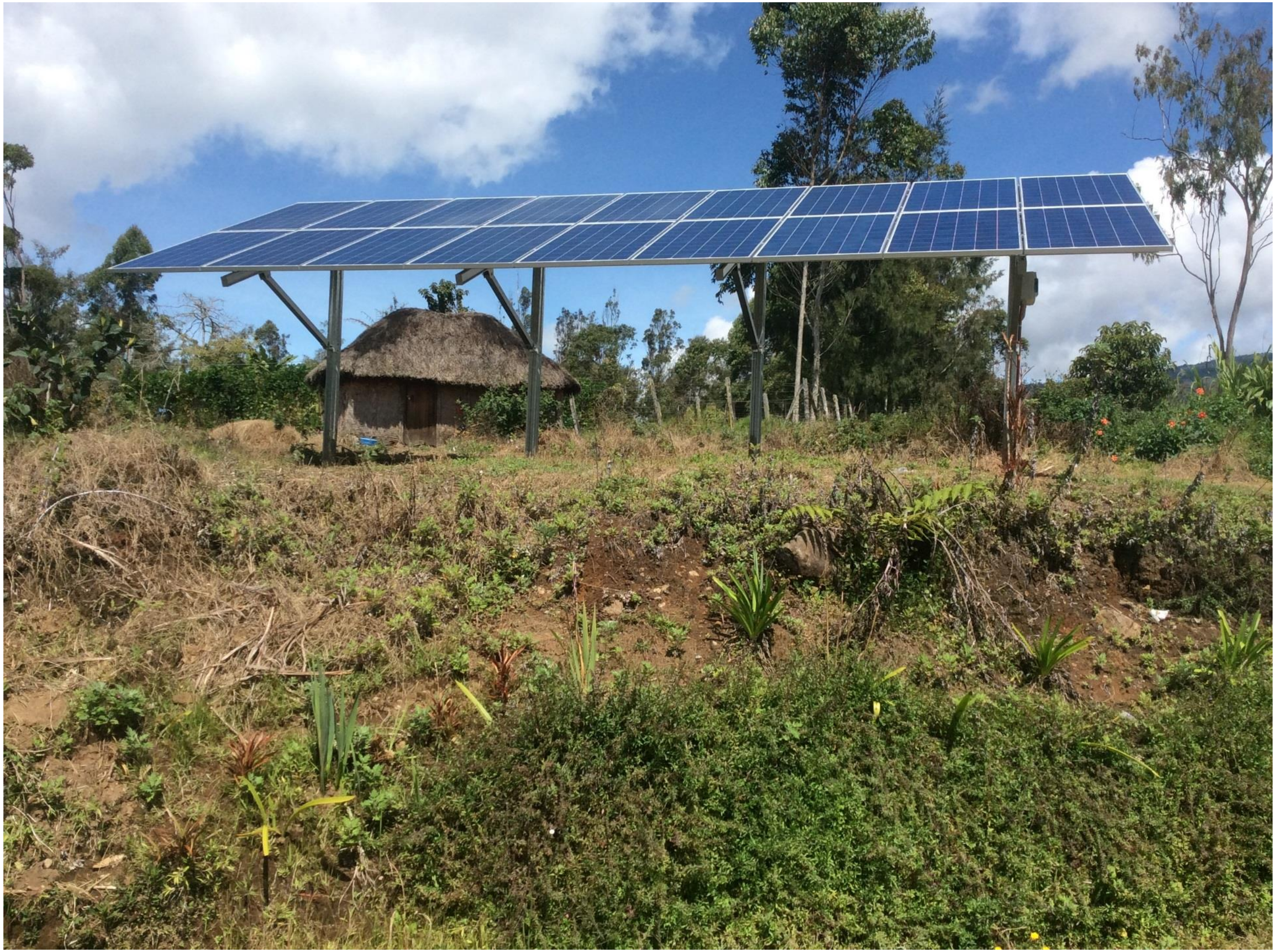

Solar panel configuration at Keripia Health Centre, Western Highlands Province, PNG
